# The anatomy of the four streams of the prefrontal cortex. Preliminary evidence from a population based high definition tractography study

**DOI:** 10.3389/fnana.2023.1214629

**Published:** 2023-10-24

**Authors:** Georgios P. Skandalakis, Jessica Barrios-Martinez, Syed Faraz Kazim, Kavelin Rumalla, Evan N. Courville, Neil Mahto, Aristotelis Kalyvas, Fang-Cheng Yeh, Constantinos G. Hadjipanayis, Meic H. Schmidt, Michael Kogan

**Affiliations:** ^1^Department of Neurosurgery, University of New Mexico Hospital, Albuquerque, NM, United States; ^2^Department of Neurosurgery, University of Pittsburgh, Pittsburgh, PA, United States; ^3^Division of Neurosurgery, Toronto Western Hospital, University Health Network, Toronto, ON, Canada

**Keywords:** cingulum, frontal slant tract, dorsal superior longitudinal fasciculus, SLF-I, uncinate fasciculus

## Abstract

The model of the four streams of the prefrontal cortex proposes 4 streams of information: motor through Brodmann area (BA) 8, emotion through BA 9, memory through BA 10, and emotional-related sensory through BA 11. Although there is a surge of functional data supporting these 4 streams within the PFC, the structural connectivity underlying these neural networks has not been fully clarified. Here we perform population-based high-definition tractography using an averaged template generated from data of 1,065 human healthy subjects acquired from the Human Connectome Project to further elucidate the structural organization of these regions. We report the structural connectivity of BA 8 with BA 6, BA 9 with the insula, BA 10 with the hippocampus, BA 11 with the temporal pole, and BA 11 with the amygdala. The 4 streams of the prefrontal cortex are subserved by a structural neural network encompassing fibers of the anterior part of the superior longitudinal fasciculus-I and II, corona radiata, cingulum, frontal aslant tract, and uncinate fasciculus. The identified neural network of the four streams of the PFC will allow the comprehensive analysis of these networks in normal and pathological brain function.

## Introduction

The pre-frontal cortex (PFC) has been suggested to serve as the central executive system of the human brain by controlling refined motor movements, goal-directed behavior, reasoning, planning, language, emotion, and memory (Wood and Grafman, 2003; Seeley et al., 2007). The medial PFC is a key component of our default mode network whereas the lateral PFC is fundamental in orchestrating high order functions (Jobson et al., 2021; Friedman and Robbins, 2022). Recently, Ben Shalom and Bonneh proposed a functional parcellation of the PFC in 4 streams, suggesting the BA8 is implicated in motor functions, BA9 for emotional processing, BA10 for memory, and BA11 for processing emotionally related sensory information (Ben Shalom and Bonneh, 2019). This model is based on data demonstrating strong functional connectivity of BA8 with BA6, BA 9 with the insula, BA10 with the hippocampus, and BA11 with the anterior temporal lobe (Shalom, 2009). Based on the functional network proposed, we hypothesize that the four streams of the PFC are subserved by connections between BA8 and BA6, BA 9 and insula, BA10 and hippocampus, and BA11 and temporal pole. To further elucidate the organization of these regions we investigated their structural connectivity using population based high definition tractography.

## Methods

We performed fiber tracking using DSI Studio software developed by FCY on a population-averaged diffusion MRI template (HP-ADMRIT) generated from diffusion MRI (dMRI) data of 1,065 human healthy subjects acquired from the Human Connectome Project (HCP) of the WashU consortium (Glasser et al., 2016; Yeh, 2022). The age range was 22–37 years, and the average age was 28.75 years. The multi-diffusion scheme included three *b*-values at 1,000, 2,000, and 3,000 s/mm^2^ and each shell had 90 sampling directions with isotropic spatial resolution at 1.25 mm, and slice thickness at 1.25 (Van Essen et al., 2013). The number of diffusion sampling directions were 90, 90, and 90, respectively. The b-table was checked by an automatic quality control routine to ensure its accuracy (Schilling et al., 2019). The diffusion data were reconstructed in the MNI space using q-space diffeomorphic reconstruction (Yeh and Tseng, 2011) to obtain the spin distribution function (Yeh et al., 2010). A diffusion sampling length ratio of 1.7 was used. The restricted diffusion was quantified using restricted diffusion imaging (Yeh et al., 2017).

Regions of interest (ROI) were assigned according to Brodmann atlas (Pijnenburg et al., 2021). ROIs of the precentral cortex included the supplementary motor area (BA6), superior frontal gyrus (BA8), medial prefrontal cortex (BA9), anterior prefrontal cortex (BA10), lateral and medial orbitofrontal cortex (BA11), insula, hippocampus, and temporal pole. We performed fiber tractography analyses to identify anatomical connections between two regions of interest following our proposed hypothesis of connection on the PFC as follows, BA 8 with BA6, BA 9 with insula, BA10 with hippocampus, BA11 with temporal pole, and BA11 with amygdala. Each region of interest was placed on the MNI space and were based on the Brodmann atlas included in the DSI Studio package. Once regions of interest were placed and anatomically verified by an anatomist. Cortical regions were assigned as “regions of interest” to allow whole brain seeding and to allow tracts to be filtered during the analyses. White matter regions were assigned as “seed” to refine fiber tractography results as this specifies the algorithm to start at this “seed” point. Tracking parameters included tracking threshold at 0, angular threshold at 0, and step size at 0 (based on default parameters). Length of fibers were based on default parameters as well (minimum length at 30 mm and maximum length at 200 mm), and these particular parameters allows to exclude tracts that are either too short (to exclude excessive u-fibers) or too long (to exclude long false continuations). In addition, we allow fiber tractography to end at 1,000,000 seeds to allow us to obtain as many results as possible. Finally, topology informed pruning was applied at 4 iterations to eliminate false continuations, a patented method described in recent publications (Yeh et al., 2019). To check for result accuracy, we followed a single-ROI approach to evaluate if fibers generated by this method will result in the same trajectories when compared to fibers obtained by pairwise tractography, and results are discussed in the results section.

## Results

**Figure 1:**
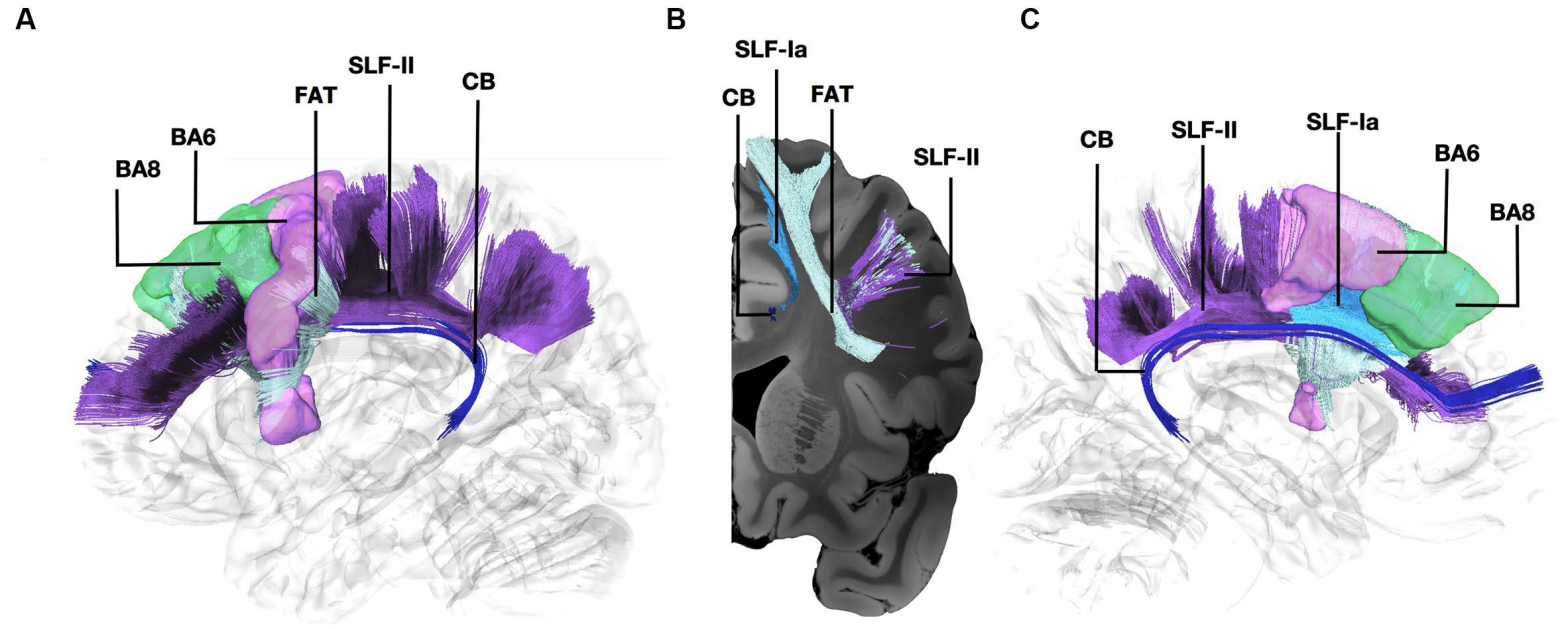
Fiber tract connectivity between BA8 and BA6 through the Frontal Aslant Tract, U-fibers, and the anterior part of the dorsal component of the Superior Longitudinal Fasciculus and Superior Longitudinal Fasciculus-II. **(A)** Lateral view demonstrating the anterior part of the left dorsal component of the superior longitudinal fasciculus in light blue, FAT in silver, and the anterior part of the superior longitudinal fasciculus II in purple interconnecting BA6 (purple) and BA8 (green) superimposed on a left hemisphere isosurface. Fibers of the cingulum are shown in dark blue. **(B)** Coronal section at the level of BA8 demonstrating the spatial relationship of the different pathways interconnecting BA6 and BA8. **(C)** Medial view demonstrating the relationship between SLF-Ia and cingulum. SLF-Ia, anterior part of the left dorsal component of the superior longitudinal fasciculus; SLF-II, Superior Longitudinal Fasciculus-II; CB, cingulum bundle; BA8, Brodmann area 8; BA6, Brodmann area 6.

**Figure 2:**
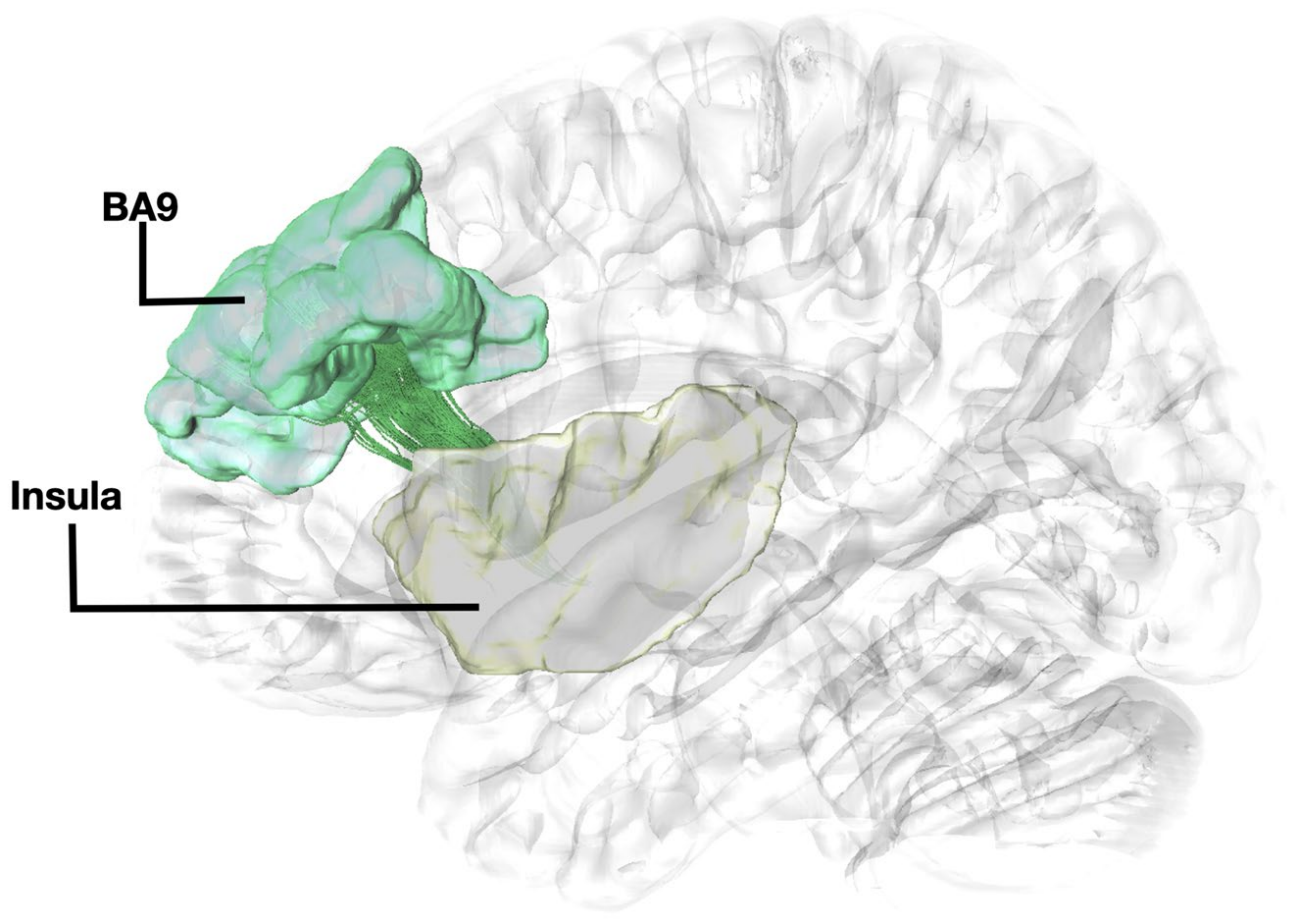
Fiber tract connectivity between BA9 and insula. Lateral view demonstrating fibers within the left corona radiata in green interconnecting the BA9 (green) with insula (posterior insular cortex) (yellow) superimposed on a left hemisphere isosurface. BA9, Brodmann area 9.

**Figure 3:**
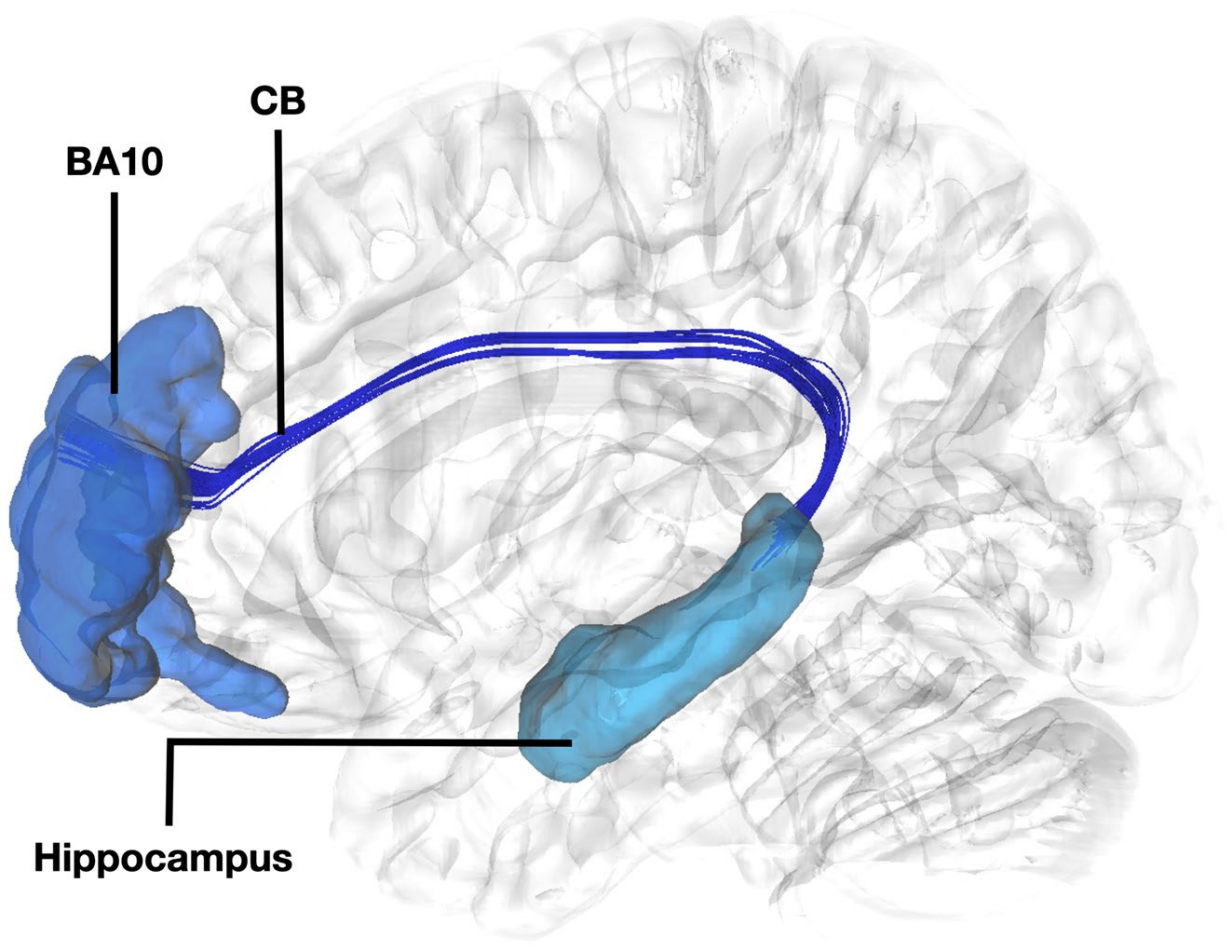
Fiber tract connectivity between BA10 and hippocampus. Lateral view demonstrating fibers of the left cingulum in dark interconnecting BA10 (dark blue) with the dorsal hippocampus (light blue) superimposed on a left hemisphere isosurface. BA10, Brodmann area 10; CB, Cingulum Bundle.

**Figure 4:**
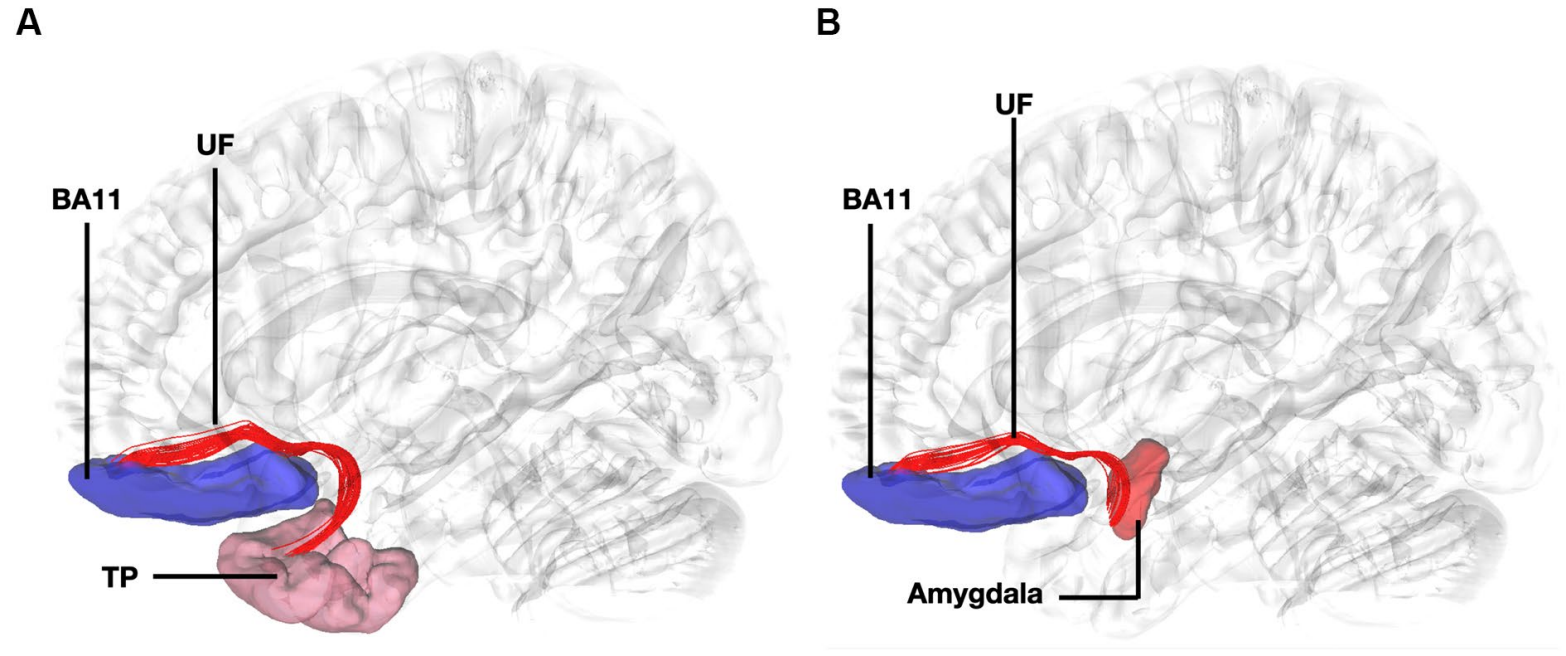
Fiber tract connectivity between BA11 and temporal pole through the uncinate fasciculus. **(A)** Lateral view demonstrating fibers of the left uncinate fasciculus in red interconnecting BA11 with temporal pole superimposed on a left hemisphere isosurface. BA11, Brodmann area 11. **(B)** Lateral view demonstrating fibers of the uncinate fasciculus interconnecting BA11 and region of amygdala superimposed on a left hemisphere isosurface. BA11, Brodmann area 11; UF, uncinate fasciculus.

Fibers running within the anterior part of the dorsal component of the superior longitudinal fasciculus (SLF-Ia) were observed interconnecting BA8 of the superior frontal gyrus (SFG) with BA6 of the pre-SMA and SMA proper. These fibers reside within the paracingulate gyrus dorsal to the body of corpus callosum. BA6 and BA8 are also interconnected with U-fibers residing within the SFG and middle frontal gyrus (MFG) as well as fibers of the superior longitudinal fasciculus II (SLF-II) (Figure 1). In addition, fibers from the frontal aslant tract (FAT) were observed connecting BA6 and BA8. Fibers interconnecting BA9 of the SFG and MFG with the insula, more specifically the posterior insular cortex, were tracked. These fibers run within the corona radiata at a rostrocaudal direction parallel to fibers of the external capsule (Figure 2). The connectivity of BA10 and hippocampus was tracked through two different fiber bundles (Figure 3). Cingulum fibers were recorded arching dorsal to the corpus callosum between BA10 and hippocampus (Figure 3). Fibers of the uncinate were tracked interconnecting BA11 with amygdala and temporal pole. Fibers implicating the amygdala were observed running medial and posterior to the fibers implicating the temporal pole. To test result accuracy, we used a single-ROI tractography approach and compared results with our original method. For example, we placed the hippocampus as a single ROI assigned as a seed to evaluate if obtained trajectories were similar to fibers obtained by pairwise tractography. Results show that fibers generated by single-ROI and two-ROI approach are the same trajectories that project from the hippocampus to BA10, which proves the pairwise tractography to be a valid method to evaluate connections of the PFC (Figure 4).

## Discussion

In this population-based tractography study, we identified direct connections of BA 8 with BA6, BA 9 with the posterior insular cortex, BA10 with the hippocampus, and BA11 with the temporal pole and amygdala through the SLF-Ia, FAT, U-Fibers, SLFII, corona radiata, and cingulum. To the best of our knowledge this is the first study demonstrating the structural connectivity of the proposed four streams of the prefrontal cortex using an HP-ADMRIT generated from dMRI data of 1,065 human healthy subjects acquired from HCP.

The connectivity between BA6 and BA8 has been demonstrated in non-human primates through tracer injections (Arikuni et al., 1988). We have recently characterized the connectivity between BA8 and BA6 through the SLF-Ia in the human brain using blunt fiber microdissections in normal human hemispheres (Komaitis et al., 2019). Our dissection results suggested that the dorsal part of the superior longitudinal fasciculus is segmented at the level of the anterior paracentral lobule in an anterior and posterior part (Komaitis et al., 2019). In line with previous anatomical studies in humans, we found the connectivity of the more lateral parts of BA8 and BA6 through the FAT, U-fibers, and the anterior segment of the SLF-II (Wang et al., 2016; Bozkurt et al., 2017).

The structural connectivity of the insula with BA9 has been previously demonstrated through a dataset of *n* = 199 subjects (Nomi et al., 2018). In addition, studies have shown connections between BA9 and BA10 and several association pathways, including cingulum and fibers from BA9 connecting to the ventral part of the insula (Petrides and Pandya, 2007). Histological studies have identified von Economo neurons both within the insula and BA9 (Fajardo et al., 2008; Allman et al., 2011). To the best of our knowledge, this is the first study reporting the trajectory, and directionality of the fibers interconnecting these regions. A tracer injection study identified major connection to BA10 including projections from parahippocampal areas, which supports our findings of fibers connecting BA10 and hippocampus (Burman et al., 2011). Furthermore, research in monkeys has demonstrated that distant regions also exhibit significant laminar similarities resulting in true anatomical connections, which has been observed in the case of projections between the BA9 and BA10 cortical areas through association fibers (Barbas, 2015). Our results show that fibers interconnecting the insula with BA9 travel within the centrum semiovale exhibiting a parallel directionality with the cortico-striatal pathways. Fibers traveling within the centrum semiovale exhibit a very complex fiber orientation pattern. Imaging results in such areas with kissing and crossing fibers are more prone to false positives (Fernandez-Miranda et al., 2012). Therefore, these results should be taken into consideration with caution. Nevertheless, results obtained by single-ROI and two-ROI approach result in the same trajectories entering the prefrontal cortex, which allows to validate our method for accuracy, and the presence of histological and imaging evidence of the connectivity of the insula with BA9, in the absence of any other fiber tracts connecting these regions support our current results.

The connectivity of BA10 and hippocampus was tracked through the cingulum. Connectivity of the BA10 and hippocampus has been reported by means of the cingulum bundle through an abundance of studies (Bubb et al., 2018; Skandalakis et al., 2020; Komaitis et al., 2022). A recent study applying diffusion tensor imaging (DTI) in children demonstrated a correlation between emotional dysregulation and increased radial diffusivity (RD), as well as decreased fractional anisotropy (FA) of the cingulum-callosal fibers, supporting the hypothesis that connecting fibers of the cingulum between BA10 and hippocampus are part of the four streams and subserving an important functional aspect of emotional regulation (Hung et al., 2020). In line with numerous fiber dissection and imaging studies in humans we showed the fibers of the uncinate interconnecting BA11 with the amygdala and temporal pole (Liakos et al., 2021). Fibers interconnecting these areas exhibit same trajectory and connectivity between humans and non-human primates (Thiebaut de Schotten et al., 2012). Furthermore, several areas of the prefrontal cortex have been shown to have similarities between human and non-human primates. However, other areas in the anterior prefrontal cortex, particularly the frontopolar region in humans, appears to be unique and not easily matched to macaque prefrontal regions, suggesting distinct cognitive capabilities in human anterior prefrontal cortex (Neubert et al., 2014). This highlights the intriguing interaction between evolutionary consistency and uniqueness within the prefrontal cortex.

DMRI provides exceptional means to study fiber tracts *in vivo*, in a fast detailed manner, allowing analysis between large populations (Yeh, 2022). Still, fiber tractography provides indirect measurements according to the diffusion of water molecules (Dyrby et al., 2018). Thus, results should be interpreted judiciously if they are not validated by cadaveric data (Yendiki et al., 2022).

## Conclusion

The 4 streams of the prefrontal cortex are subserved by a structural neural network involving fibers of the anterior part of the superior longitudinal fasciculus-I, superior longitudinal fasciculus-II, corona radiata, uncinate fasciculus, frontal aslant tract, and U-fibers. The identified neural network of the four streams of the PFC will allow a more comprehensive analysis of these networks in normal and pathological brain function.

## Data availability statement

The raw data supporting the conclusions of this article will be made available by the authors, without undue reservation.

## Ethics statement

Ethical approval was not required for the studies involving humans because the patients/participants provided their written informed consent to participate in this study. The studies were conducted in accordance with the local legislation and institutional requirements. The participants provided their written informed consent to participate in this study.

## Author contributions

GS, F-CY, KR, SK, CH, AK, and MK: concept and design. GS, F-CY, KR, SK, NM, AK, EC, CH, MS, and MK: data acquisition and analysis. GS, AK, F-CY, CH, MS, and MK: supervision. GS, KR, SK, EC, and NM: drafting. GS, F-CY, KR, SK, AK, CH, MS, and MK: critical review and editing. All authors reviewed and approved the final manuscript.
